# Experimental model of lung donors with hemorrhagic shock treated with hypertonic saline solution and ex-vivo evaluation with lung perfusion system

**DOI:** 10.1186/1749-8090-8-S1-O237

**Published:** 2013-09-11

**Authors:** NA Nepomuceno, MS Samano, KAO Braga, LM Ruiz, EZS Pato, BKS Hirata, PM Pêgo-Fernandes, FB Jatene

**Affiliations:** 1 Thoracic Surgery Department, Heart Institute (InCor), Hospital das Clínicas da Faculdade de Medicina da Universidade de São Paulo, São Paulo, Brazil; 2 Cardiovascular Surgery Department, Heart Institute (InCor), Hospital das Clínicas da Faculdade de Medicina da Universidade de São Paulo, São Paulo, Brazil

## Background

One of the main reasons for discharge lung donors is pulmonary edema. It may be associated to hemodynamic shock frequently present among organ donors. There is no experience using hypertonic saline solution among lung donors with hypovolemic shock. The aim of this study is to evaluate hypertonic solution in pulmonary function of lung donor model with hypovolemic shock using the ex-vivo lung perfusion system.

## Methods

Eighty Sprague-Dawley rats were anesthetized. Femoral artery was cannulated and used to register mean artery pressure (MAP). They were then randomized in four groups (Sham, Shock, Saline or Hypertonic). Hypovolemic shock was induced with blood retrieval until reduction of MAP to 40mmHg and maintained for 60 minutes. Hypertonic group received hypertonic solution (4mL/Kg) and Saline group 33ml/Kg. All groups were treated as lung donors and perfused with Perfadex (20mL/Kg). Forty heart-lung blocks were connected to ex-vivo perfusion system and perfused for 60 minutes. Another forty blocks were analyzed without ex-vivo perfusion. MAP, pulmonary resistance, compliance, oxygen capacity, wet-to-dry ratio, TNF-α, IL-1ß, IL-6 and neutrophils were analyzed.

## Results

The experimental model of shock was feasible, reproducible and hypertonic solution recovered normal MAP levels. However there were no difference in pulmonary compliance, resistance and oxygen capacity. Although Shock group showed higher levels of IL-6 and IL-1ß, there were no difference in these cytokines and TNF-α among all groups. The ex-vivo lung perfusion caused elevation of all cytokines immunoexpression.

## Conclusion

Hypertonic Saline Solution does not improve pulmonary function or reduce inflammatory markers after hypovolemic shock in animal model of lung donors.

